# Long‐term changes in occurrence, relative abundance, and reproductive fitness of bat species in relation to arrival of White‐nose Syndrome in West Virginia, USA

**DOI:** 10.1002/ece3.7991

**Published:** 2021-08-04

**Authors:** Catherine Johnson, Donald J. Brown, Chris Sanders, Craig W. Stihler

**Affiliations:** ^1^ Monongahela National Forest U.S. Forest Service Elkins West Virginia USA; ^2^ Region 1 National Park Service Narragansett Rhode Island USA; ^3^ School of Natural Resources West Virginia University Morgantown West Virginia USA; ^4^ Northern Research Station U.S.D.A. Forest Service Parsons West Virginia USA; ^5^ Sanders Environmental Inc. Bellefonte Pennsylvania USA; ^6^ West Virginia Division of Natural Resources (retired) Elkins West Virginia USA

**Keywords:** bats, body condition, *Corynorhinus townsendii virginianus*, disease, *Eptesicus fuscus*, generalized additive model, *Lasiurus borealis*, *Myotis lucifugus*, *Myotis septentrionalis*, *Perimyotis subflavus*, reproduction, summer, white‐nose syndrome

## Abstract

White‐nose syndrome (WNS) is a disease caused by the fungus *Pseudogymnoascus destructans* which has resulted in the deaths of millions of bats across eastern North America. To date, hibernacula counts have been the predominant means of tracking the spread and impact of this disease on bat populations. However, an understanding of the impacts of WNS on demographic parameters outside the winter season is critical to conservation and recovery of bat populations impacted by this disease. We used long‐term monitoring data to examine WNS‐related impacts to summer populations in West Virginia, where WNS has been documented since 2009. Using capture data from 290 mist‐net sites surveyed from 2003 to 2019 on the Monongahela National Forest, we estimated temporal patterns in presence and relative abundance for each bat species. For species that exhibited a population‐level response to WNS, we investigated post‐WNS changes in adult female reproductive state and body mass. *Myotis lucifugus* (little brown bat), *M*. *septentrionalis* (northern long‐eared bat), and *Perimyotis subflavus* (tri‐colored bat) all showed significant decreases in presence and relative abundance during and following the introduction of WNS, while *Eptesicus fuscus* (big brown bat) and *Lasiurus borealis* (eastern red bat) responded positively during the WNS invasion. Probability of being reproductively active was not significantly different for any species, though a shift to earlier reproduction was estimated for *E*. *fuscus* and *M*. *septentrionalis*. For some species, body mass appeared to be influenced by the WNS invasion, but the response differed by species and reproductive state. Results suggest that continued long‐term monitoring studies, additional research into impacts of this disease on the fitness of WNS survivors, and a focus on providing optimal nonwintering habitat may be valuable strategies for assessing and promoting recovery of WNS‐affected bat populations.

## Graphical abstract

We used long‐term monitoring data to examine impacts from white‐nose syndrome on summer bat populations in West Virginia. The little brown bat, northern long‐eared bat, and tri‐colored bat all showed significant decreases in survey presence and relative abundance during and following the introduction of WNS, while the big brown bat and eastern red bat responded positively to the WNS invasion. We also investigate post‐WNS changes in adult female reproduction and body mass for those species in the study area.

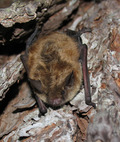

## 1 INTRODUCTION

**FIGURE 1:**
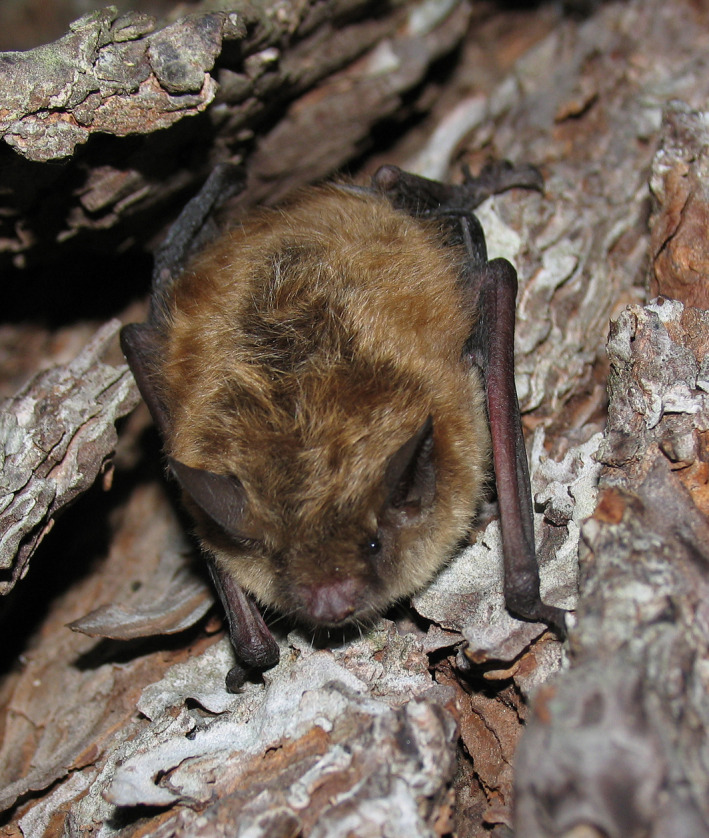
An adult *Myotis lucifugus* (little brown bat), released on a tree after mist‐net capture in Grant County, West Virginia, USA. Photograph used with permission from Keith Christenson

White‐nose syndrome (WNS) is a deadly disease caused by the fungus *Pseudogymnoascus destructans* (*Pd*) which affects hibernating bats. WNS was first discovered in a cave in New York in 2007; by 2012, it had been confirmed as present in caves across most of the eastern United States and was estimated to have killed 5.7–6.7 million bats (US Fish & Wildlife Service, 2012). Since then, the disease and bat death toll has spread across much of North America, with new species being affected as the disease crosses into different geographic ranges. Population impacts have varied by species, with some of the most heavily impacted bat species presently including the endangered *Myotis sodalis* (Indiana bat), *Perimyotis subflavus* (tri‐colored bat), and *Myotis lucifugus* (little brown bat; Figure 1). In West Virginia, hibernacula counts for these species have declined by ~97%, 90%, and 97%, respectively, since WNS was first detected in the state in 2009 (West Virginia Division of Natural Resources [WVDNR], unpublished data). Hibernacula counts have been the predominant means of tracking the spread and impact of this disease (e.g., Frick, Pollock, et al., 2010; Langwig et al., 2012; Powers et al., 2015; Turner et al., 2011). However, some species, such as *Myotis septentrionalis* (northern long‐eared bat), are also highly vulnerable to WNS but are less conspicuous in hibernacula, making their populations difficult to track with winter counts. Monitoring of populations outside the hibernacula season may provide the best means of tracking changes in these populations.

Monitoring of summer bat populations using either acoustic or capture surveys provides a means to assess relative changes in population numbers over time. Several studies have detected declines in *Myotis* summer populations following WNS arrival in an area (e.g., Brooks, 2011; Dzal et al., 2011; Moosman et al., 2013; Reynolds et al., 2016) that often track those seen in hibernacula. As such, the impacts of WNS on population trends of several species are well documented during both winter and summer across geographic areas where *Pd* has been present for many years. However, there is a paucity of information regarding the impacts to reproduction and body condition, particularly in the natural environment and outside of hibernation. Higher body fat stores in hibernating bats are likely advantageous for both over‐winter survival and successful reproduction the following year (Cheng et al., 2019; Kunz et al., 1998); this is especially true for bats infected with *Pd*, as they are subject to more frequent and longer arousal periods and resultant increases in body fat depletion during hibernation (Lilley et al., 2017; Reeder et al., 2012). Higher body fat in early winter could reduce WNS mortality by 58%–70%, providing higher energy stores to be depleted throughout hibernation to deal with the demands of fighting *Pd* infection during that period (Cheng et al., 2019). Furthermore, *M*. *lucifugu*s appears to have evolved mechanisms to conserve fat reserves during hibernation by maintaining normal torpor patterns in the presence of *Pd* (Frank et al., 2019). In addition to the potential loss of fat reserves during hibernation, energy demands for *Pd*‐infected individuals upon emergence from hibernacula are particularly high as they attempt to recover from infection‐related tissue (wing) damage with a potential associated reduction in flight and, thus, foraging efficiency (Fuller et al. 2020).

Over time, steep declines in adult survival as a result of WNS may be ameliorated in different ways, such as developing resistance to pathogen infection or physiological adaptations in survivors (Frank et al., 2019; Langwig et al., 2017). However, population‐level impacts of WNS likely extend well beyond those reflected by adult mortality rates, as WNS‐infected individuals emerging from hibernacula are faced with increased energetic demands to cope with healing of skin and wing damage and heightened stress levels (Davy et al., 2017; Meierhofer et al., 2018), in addition to the normally high energy requirements of bats during spring emergence. Given the high energetic cost to females of gestation and raising pups, it is likely that there is some energetic trade‐off between adult annual survival and successful reproduction for WNS‐affected bats emerging from hibernacula. Life history theory predicts that in organisms with high adult survival, such as bats, females should forego reproduction and allocate resources to their own maintenance and survival when resources are limited and the probability of survival of their young is particularly low (Barclay et al., 2004). Thus, it is possible that WNS‐affected females facing the high energetic cost of recovery upon emergence might forego reproduction and instead allocate resources to recovery and increasing body mass prior to entering hibernacula the following winter.

A better understanding of the fitness of WNS‐affected individuals and populations requires an assessment of demographic parameters such as age, sex, reproductive state, and body condition, collected in the field as part of capture surveys. Such detailed demographic data are critical to our understanding of how WNS affects individuals and populations that do survive infection, enabling more accurate population viability analyses and informing potential recovery strategies for species already impacted. Unfortunately, long‐term capture data are rarely available for bat species and even fewer are available for assessing bat communities, though pre‐ and post‐WNS capture data have been used to detect declines in *Myotis* species in New Hampshire (Moosman et al., 2013), Indiana (Pettit & O’Keefe, 2017), Kentucky (Thalken et al., 2018), and the Great Smoky Mountains of North Carolina and Tennessee (O’Keefe et al., 2019). When such data do exist, the numbers are generally too small to assess more than population trends. Analyses of demographic parameters such as changes in reproductive state or body condition pre‐ and post‐WNS are especially difficult since numbers of those species most‐affected decline so precipitously that sample sizes post‐WNS are very small (Lacki et al., 2015).

Understanding the condition and reproductive status of WNS‐affected bats that survive hibernation can also help illuminate the trade‐offs that may affect persistence of populations post‐WNS, inform vulnerability assessments for species most‐affected, and help to focus conservation efforts in habitats used outside the hibernation season. Here, we present data from a long‐term summer bat monitoring program at the Monongahela National Forest (MNF) in West Virginia used to assess changes in the bat community and population parameters of multiple species in the MNF pre‐ and post‐WNS, including an analysis of changes in body condition as well as reproductive status and timing in response to WNS.

We predicted that summer populations of WNS‐vulnerable species in our study area would reflect the steep declines observed in hibernacula counts for species such as *M*. *lucifugus* and *P*. *subflavus* in the region (Powers et al., 2015; Turner et al., 2011; WVDNR, unpublished data) and the general lack of *M*. *septentrionalis* captures and recordings across the summer landscape in the northeast post‐WNS (Moosman et al., 2013; Reynolds et al., 2016). Species breeding in the study area that are not considered to be vulnerable to WNS were expected to remain relatively stable, with the exception of *Corynorhinus townsendii virginianus* (Virginia big‐eared bat), which had been steadily increasing prior to WNS invasion as a result of conservation actions (Stihler, 2011) and was predicted to continue on that track. There are few published data available to inform predictions on reproductive status and body condition post‐WNS. However, given the energetic costs faced by *Pd*‐infected individuals emerging from hibernacula (Fuller et al., 2020; Meierhofer et al., 2018), we expected to see adverse impacts to WNS‐vulnerable species reflected in reproduction, body condition, or both.

## 2 METHODS

### 2.1 Study area

This study was conducted across the Monongahela National Forest (MNF), West Virginia, USA, which encompasses 372,715 ha of federally owned land within a 687,966 ha proclamation boundary (US Forest Service, 2006). The MNF stretches across a latitudinal range of nearly 200 km and lies within two ecoregions, the Central Appalachians and the Ridge and Valley. Elevation ranges from 275–1,480 m and precipitation ranges from ~75 cm/year in the eastern section of the MNF to 115–150 cm/year across the remainder of the forest. The MNF is composed primarily of 70‐ to 100‐year‐old forest stands with high regional tree diversity and four major forest zones (mixed mesophytic, northern hardwoods, red spruce, and dry oaks).

The proclamation boundary of the MNF overlays many bat hibernacula, including Hellhole Cave in Pendleton County, which is the largest bat hibernaculum in West Virginia and designated critical habitat for the endangered *M*. *sodalis* and *C.t*. *virginianus*. White‐nose syndrome was first detected in West Virginia in January–February 2009 at four caves in Pendleton County, including one cave located within the MNF. By early 2010, WNS was detected in five additional counties and by the winter of 2010/11, 13 of the 14 caves (92.9%) monitored across the MNF’s landscape were WNS‐positive, with the remaining cave considered suspect (WVDNR, unpublished data).

### 2.2 Data collection

**FIGURE 2:**
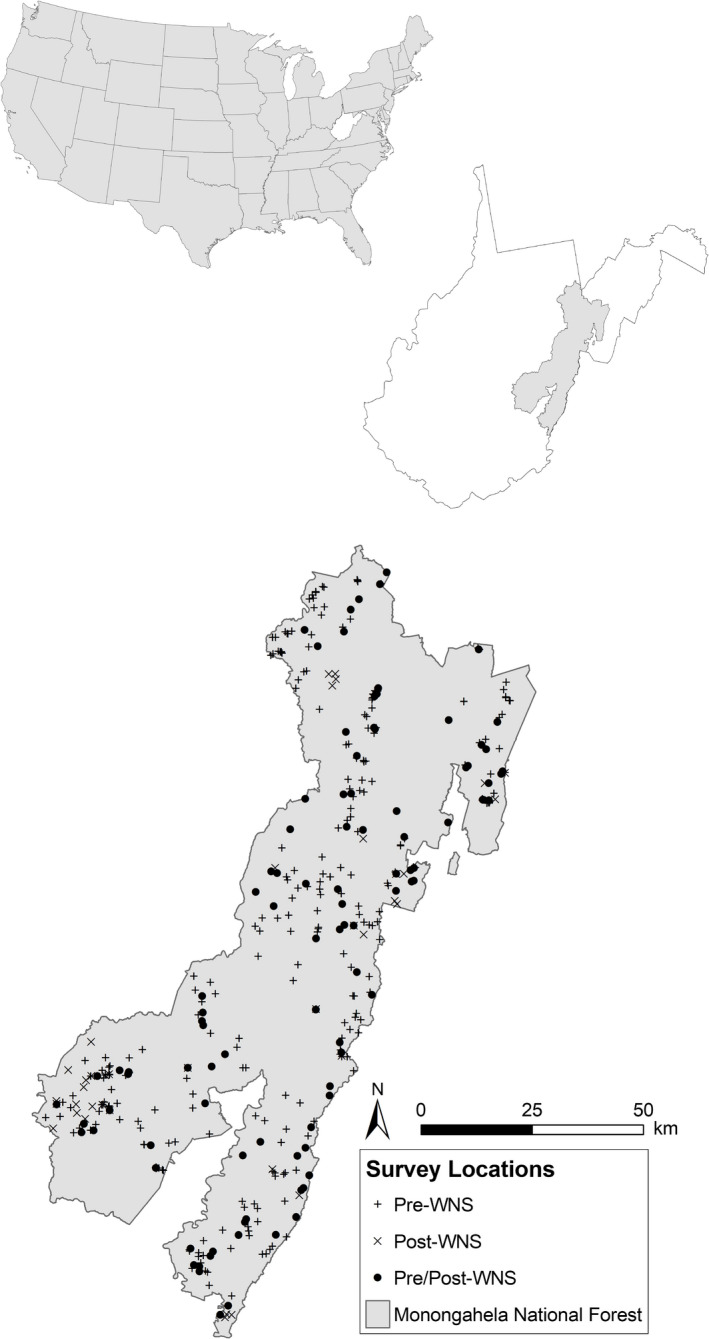
Bat mist‐net survey locations on the Monongahela National Forest (MNF), West Virginia, USA. From 2003 to 2019, 1,248 mist‐net surveys were conducted across 290 sites. Sites are grouped as preintroduction of white‐nose syndrome only (Pre‐WNS; 2003–2008), postintroduction of WNS only (Post‐WNS; 2009–2019), and both pre‐ and postintroduction of WNS (Pre/Post‐WNS)

The MNF has conducted annual summer bat mist‐netting since 1997. From 2003 to 2019, mist‐net surveys were conducted across 290 sites (Figure 2), including 60 long‐term monitoring sites surveyed on a rotating basis using consistent methodology (i.e., location, net‐sets, time of year) and level of effort. Mist‐net sites were distributed across locations representative of all primary habitat types on the MNF, ranging from field and early successional habitat to oak‐pine and northern hardwood forest, and were selected near potential bat travel corridors or over areas considered to have a high potential to be used by bats for foraging or drinking.

Mist‐net poles and nets were set up prior to dusk and nets deployed at dusk. A minimum of two net locations were used at each site (≥30 m apart), and nets were operated for a minimum of 5 hr each night. Net‐set heights and lengths ranged from 2.6 m (single‐high) to 10.4 m (quadruple‐high) and from 3 to 18 m, respectively, depending on the physical characteristics of the site. Nets were checked every 8–10 min while deployed.

Nets were operated for 2 nights/year at each site. When inclement weather prevented completion of a full survey at a given site, additional surveys were conducted until 2 full survey nights were completed. Individual bat data from partial survey nights were included in reproductive state and body condition analyses, but partial survey nights were excluded from presence and relative abundance analyses so that sampling effort was consistent across sites. At least 20 sites were surveyed each year (range = 20–58 sites/year, mean = 37.9) at locations distributed across the MNF. Individual sites were surveyed from 1 to 10 years over the 17‐year study period (mean = 2.1 years/site). Of the 290 survey sites, 250 were surveyed pre‐WNS (2003–2008) and 135 were surveyed from 2009 to 2019; 96 sites (33%) were surveyed during both time periods, with 58 of those sites having at least 4 years of survey data.

Data recorded for each bat captured included: species, sex, age, mass (g), reproductive condition, and forearm length (mm). Age (juvenile or adult) was determined by degree of ossification of the finger joints (Kunz & Anthony, 1982), and reproductive condition was recorded as pregnant, lactating, postreproductive, or nonreproductive for females and scrotal or nonreproductive for males (Racey, 1988). Surveys followed U.S. Fish and Wildlife Service WNS protocols (e.g., U.S. Fish & Wildlife Service, 2018) and handling guidelines for bats, Monongahela National Forest protocol, and WV Division of Natural Resources permit requirements. Bat morphological data were collected following the Mammal Collectors' Manual (Nagorsen & Peterson, 1980).

Eleven bat species were captured including nine species that breed in West Virginia: *C*.*t. virginianus*, *Eptesicus fuscus* (big brown bat), *Lasionycteris noctivagans* (silver‐haired bat), *Lasiurus borealis* (eastern red bat), *Lasiurus cinereus* (hoary bat), *Myotis leibii* (eastern small‐footed myotis), *M*. *lucifugus*, *M*. *septentrionalis*, *M*. *sodalis* (Indiana bat), *Nycticeius humeralis* (evening bat), and *P*. *subflavus*. For this study, we excluded *M*. *sodalis* due to model convergence problems caused by a small sample size (56 captures) and three migratory species with low captures (*L. cinereus* [192 captures], *L*. *noctivagans* [92 captures], and *N*. *humeralis* [1 capture]). We also removed 60 capture records that were only identified to the genus *Myotis*.

### 2.3 Statistical analyses

#### 2.3.1 Presence and relative abundance

We used two‐part zero‐inflated Poisson generalized additive models (ZIPGAM) to estimate temporal patterns in presence and relative abundance (i.e., site detection and count, respectively) for each bat species from 2003 to 2019 (Wood et al., 2016). We chose zero‐inflated models because the survey data contained a large proportion of zero counts (i.e., 56% of sites across all species and survey years), and the count data distributions did not satisfy assumptions of standard Poisson or negative binomial distributions. We chose generalized additive models rather than generalized linear models to allow for potentially complex trend dynamics, which is common for long‐term monitoring data (e.g., Fedy & Aldridge, 2011; Fewster et al., 2000). The two‐part models included binary (presence–absence) and continuous (count) states, where the binary state was modeled using a binomial distribution and the continuous state was modeled using a truncated Poisson distribution (Cunningham & Lindenmayer, 2005). To estimate the influence of year on presence and relative abundance, we included year as a smoothed continuous predictor for both the binary and continuous states. The smooth parameter was modeled using restricted maximum likelihood with Laplace approximation, which has been shown to reduce undersmoothing compared with generalized cross‐validation (Reiss & Ogden, 2009; Wood, 2011).

To identify time periods of significant population increases and decreases, we computed 200 first‐order derivatives (i.e., slope of the tangent line) across the 17‐year period using the finite difference method (Simpson, 2018), and considered periods of significant change to be those in which the 95% point‐wise confidence intervals did not overlap 0. We assessed significance of model terms using Wald tests with α = 0.05 (Zuur et al., 2009). We examined model assumptions using residual diagnostic plots (Jones & Wrigley, 1995; Zuur et al., 2014), as well as ensured model convergence was reached and that the smoothing parameter was not overfit based on the k‐index test (Wood, 2017). We examined model performance by comparing modeled trend estimates to annual summaries of survey data (i.e., proportion of sites with detections and mean count at sites with detections) to ensure the models reflected the patterns in the observation data.

#### 2.3.2 Reproductive state and body condition

We investigated changes in adult female reproductive state and body condition for species that exhibited a population‐level response to WNS (*E*. *fuscus*, *L. borealis*, *M*. *lucifugus*, *M. septentrionalis*); *P*. *subflavus* was excluded because of a low post‐WNS sample size. We grouped the observation years into two categories representing invasion status: pre‐WNS (2003–2008) and post‐WNS (2011–2019), excluding data from the primary invasion years (2009–2010). We did not model year‐specific dynamics because of low annual sample sizes for WNS‐impacted species following invasion of the disease.

For the reproductive state models, we included day of year as the null model, and assessed whether WNS invasion status was a supported additive predictor using likelihood‐ratio tests (α = 0.05; Zuur et al., 2009). We modeled probability of being nonreproductive or reproductive to investigate changes in reproductive potential. We also modeled probability of being in four states: nonreproductive, pregnant, lactating, and postreproductive, to investigate potential shifts in reproductive timing. We used logistic regression for the two‐state models and ordinal regression for the four‐state models (Agresti, 2007). We examined model assumptions and performance using residual diagnostic plots and receiver operating characteristic (ROC) curves (Agresti, 2007; Pardoe & Cook, 2002).

We used body mass rather than a body condition index (BCI) to assess body condition for bats in this study. While BCI (body mass divided by forearm length) has often been used to assess body condition in bats, McGuire et al. (2018) found that forearm length does not accurately correct for intraspecific variation in body size and that body mass alone is a better predictor of fat mass in bats. For the body mass models, we included reproductive state as the null model, and assessed whether WNS invasion status was a supported interaction predictor of body mass using likelihood‐ratio tests (α = 0.05). This model estimates the influence of WNS on body mass of bats in each reproductive state. We used linear regressions with a Gaussian distribution for the body mass analyses (Zuur et al., 2009). We performed model validation using residual diagnostic plots and removed 8 outlier observations among the species to satisfy assumptions of normality and homoscedasticity.

We conducted all analyses using program R (version 3.6.3). We constructed the ZIPGAM models using the function ‘ziplss’ in package mgcv (version 1.8‐28), and computed first‐order derivatives and confidence intervals using the function ‘confint.fderiv’ in package gratia (version 0.2‐8). We constructed the linear and logistic regression models using the stats package in base R, and the ordinal regression models using the function ‘ocat’ in package mgcv. We computed ROC curves for logistic regressions using the package LogisticDx (version 0.2). We created graphs using the packages ggplot2 (version 3.2.1), cowplot (version 1.0.0), and sjplot (version 2.8.7).

## 3 RESULTS

### 3.1 Presence and relative abundance

Across all monitoring years, we captured a total of 159 *C.t*. *virginianus*; 1,311 *E. fuscus*; 1,334 *L*. *borealis*; 151 *M*. *leibii*; 1,813 *M*. *lucifugus*; 4,069 *M*. *septentrionalis*; and 502 *P. subflavus*. Mean annual captures per site across all focal species was 18.0 (±2.8) pre‐WNS (2003–2008) and 8.6 (±3.1) post‐WNS (2011–2019). Mean annual percentage of total captures increased from pre‐ to post‐WNS for *C.t*. *virginianus* (0.5 [±0.5]% to 5.8 [±4.8]%), *E*. *fuscus* (9.7 [±4.3]% to 33.3 [±10.3]%), *L. borealis* (9.3 [±3.1]% to 33.3 [±5.7]%), and *M*. *leibii* (0.8 [±0.6]% to 4.9 [±2.1]%) and decreased for *M*. *lucifugus* (24.6 [±6.6]% to 1.9 [±1.0]%), *M. septentrionalis* (48.8 [±9.3]% to 18.8 [±10.7]%), and *P*. *subflavus* (6.2 [±2.4]% to 2.0 [±1.5]%). Year was a significant predictor for all models. Deviance explained ranged from 8.6% to 16.3% for species negatively impacted by WNS, and from 2.6% to 6.2% for the remaining species.

**FIGURE 3:**
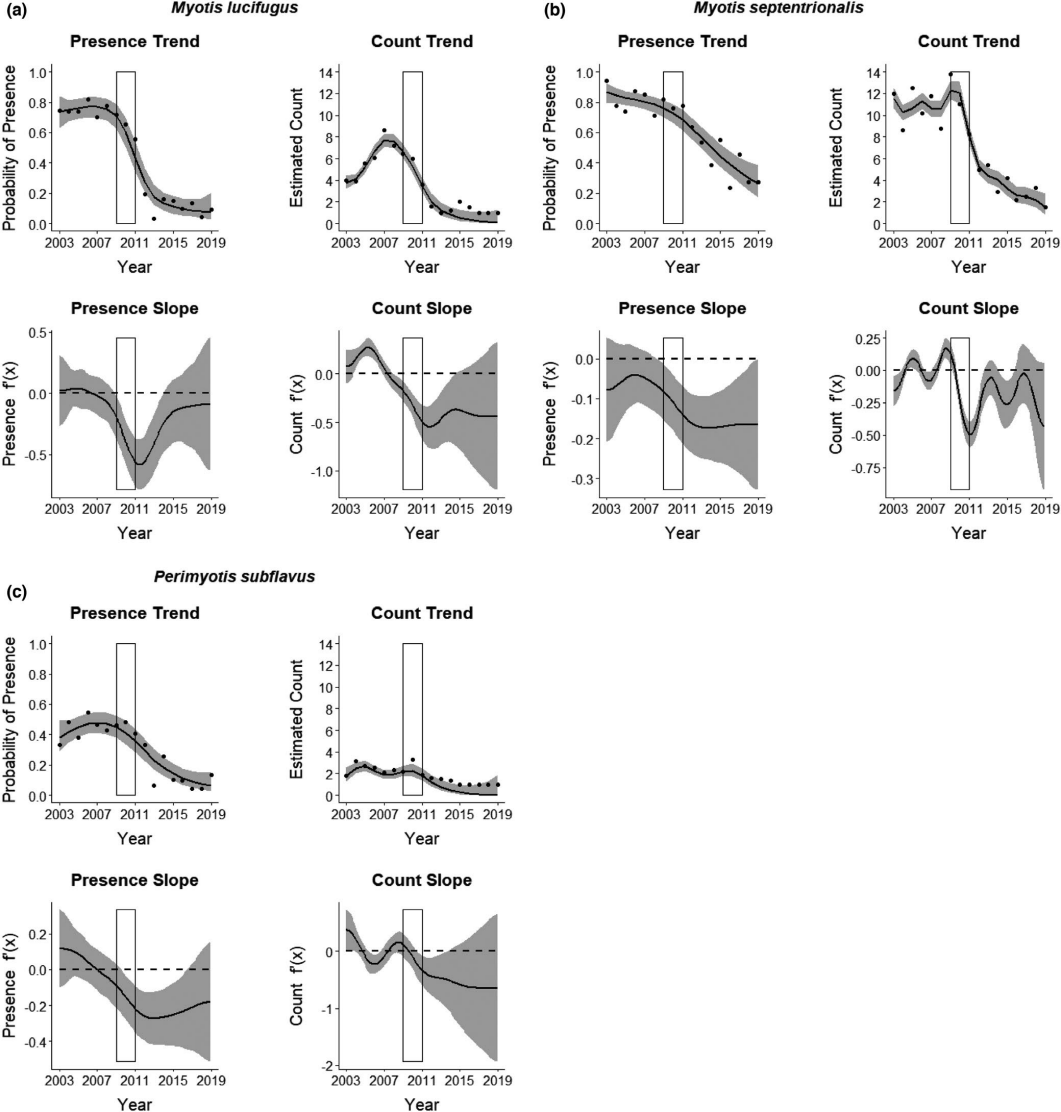
Presence and count trends from 2003 to 2019 for bat species in the Monongahela National Forest (MNF), West Virginia, USA, that responded negatively to invasion of white‐nose syndrome (WNS), including (a) *Myotis lucifugus* (little brown bat; *n* = 1,813 captures), (b) *Myotis septentrionalis* (northern long‐eared bat; *n* = 4,069 captures), and (c) *Perimyotis subflavus* (tri‐colored bat; *n* = 502 captures). We estimated bat trends using annual mist‐net survey data and zero‐inflated Poisson generalized additive models (ZIPGAM), with year included as a smoothed continuous predictor. At least 20 sites were surveyed annually, with two surveys per site within years. For each species, the upper graphs display the probability of presence and estimated count for occupied sites (black lines) and their 95% confidence intervals (gray bands), and the bottom graphs display the rate of change (i.e., slope of the tangent line) for the presence and count estimates. A positive slope indicates the population is increasing relative to the slope in the previous time step, and vice versa. The points on the presence trend graphs represent the annual mean proportion of sites with observed presence. The points on the count trend graphs represent the annual mean of the total count per site at observed occupied sites (note the lower bound is 1). The rectangle in each graph encompasses the years of observed spread of WNS across the MNF (2009–2011)

The population trend models indicated three of the eight modeled species were heavily impacted by WNS, including *M*. *lucifugus*, *M*. *septentrionalis*, and *P*. *subflavus*, with all three showing significant decreases in survey presence and relative abundance during and following the introduction of WNS (Figure 3). The decline was greatest for *M*. *lucifugus*, with estimated probability of presence at sites decreasing from a high of 0.771 in 2006 to 0.064 in 2019. Probability of presence for *M*. *septentrionalis* also decreased substantially, from a high of 0.868 in 2003 to 0.262 in 2019. Probability of presence for *P*. *subflavus* decreased from a high of 0.472 in 2006 to 0.047 in 2019. None of the three species indicated presence or relative abundance was beginning to rebound by 2019 (Figure 3).

**FIGURE 4:**
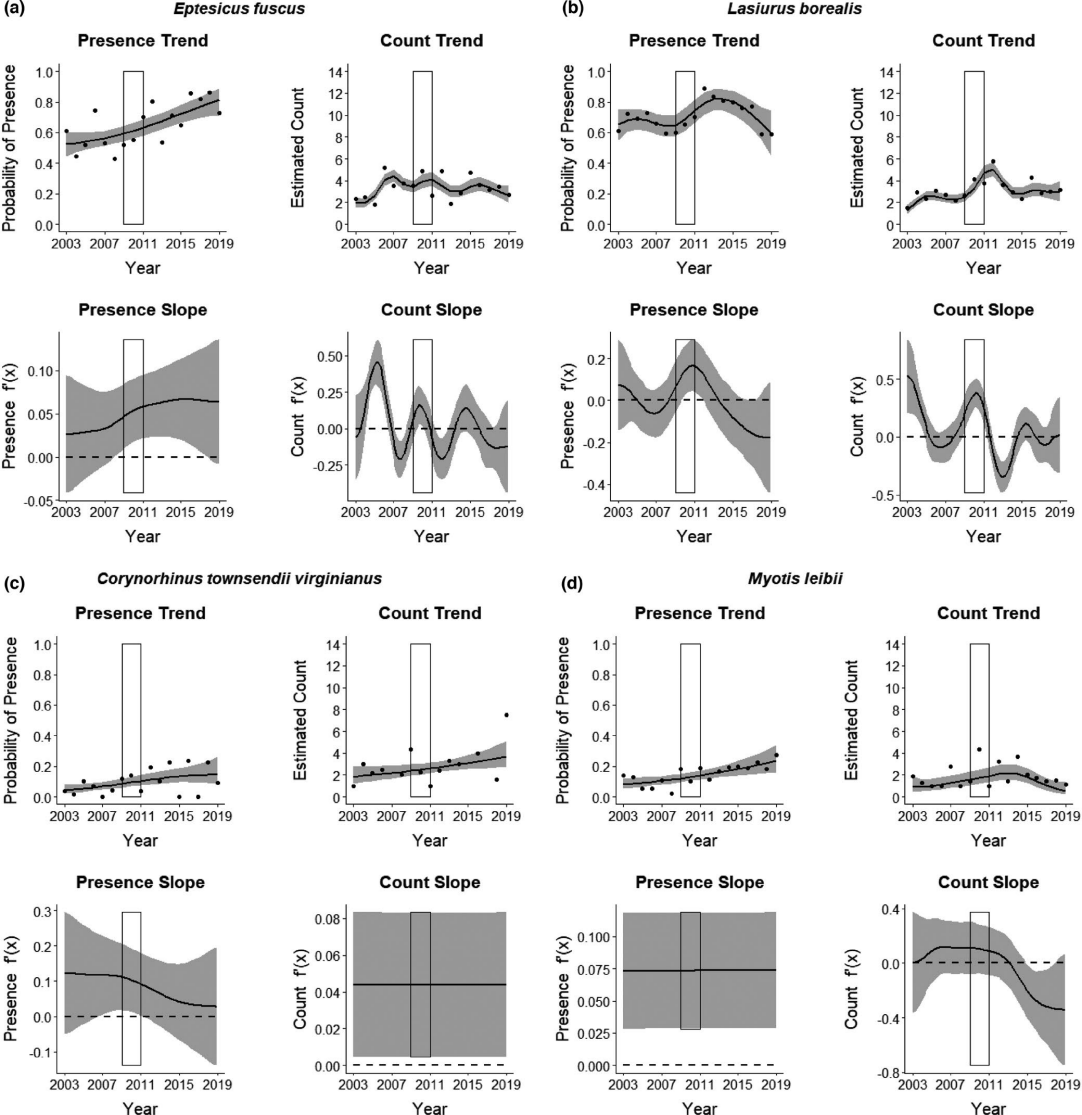
Presence and count trends from 2003 to 2019 for bat species in the Monongahela National Forest (MNF), West Virginia, USA, that responded positively, including (a) *Eptesicus fuscus* (big brown bat; *n* = 1,311 captures), (b) *Lasiurus borealis* (eastern red bat; *n* = 1,334 captures); or showed no clear response, including (c) *Corynorhinus townsendii virginianus* (Virginia big‐eared bat; *n* = 159 captures), and (d) *Myotis leibii* (eastern small‐footed myotis; *n* = 151 captures) to invasion of white‐nose syndrome (WNS). We estimated bat trends using annual mist‐net survey data and zero‐inflated Poisson generalized additive models (ZIPGAM), with year included as a smoothed continuous predictor. At least 20 sites were surveyed annually, with two surveys per site within years. For each species, the upper graphs display the probability of presence and estimated count for occupied sites (black lines) and their 95% confidence intervals (gray bands), and the bottom graphs display the rate of change (i.e., slope of the tangent line) for the presence and count estimates. A positive slope indicates the population is increasing relative to the slope in the previous time step, and vice versa. The points on the presence trend graphs represent the annual mean proportion of sites with observed presence. The points on the count trend graphs represent the annual mean of the total count per site at observed occupied sites (note the lower bound is 1). The rectangle in each graph encompasses the years of observed spread of WNS across the MNF (2009–2011)

Two species, *E*. *fuscus* and *L. borealis*, responded positively during the WNS invasion. *Eptesicus fuscus* increased in probability of presence (Figure 4a) and *L. borealis* increased in both probability of presence and estimated count at occupied sites (Figure 4b). Following the invasion period, probability of presence continued to increase through 2019 for *E*. *fuscus*, but returned to pre‐WNS levels by 2019 for *L. borealis*. The remaining bat species assessed (*C.t*. *virginianus* and *M*. *leibii*) did not exhibit a strong response to introduction of the disease (Figure 4c,d). However, estimated count for *C.t*. *virginianus* and probability of presence for *M*. *leibii* increased over the 17‐year monitoring period.

### 3.2 Reproductive state and body mass

**FIGURE 5:**
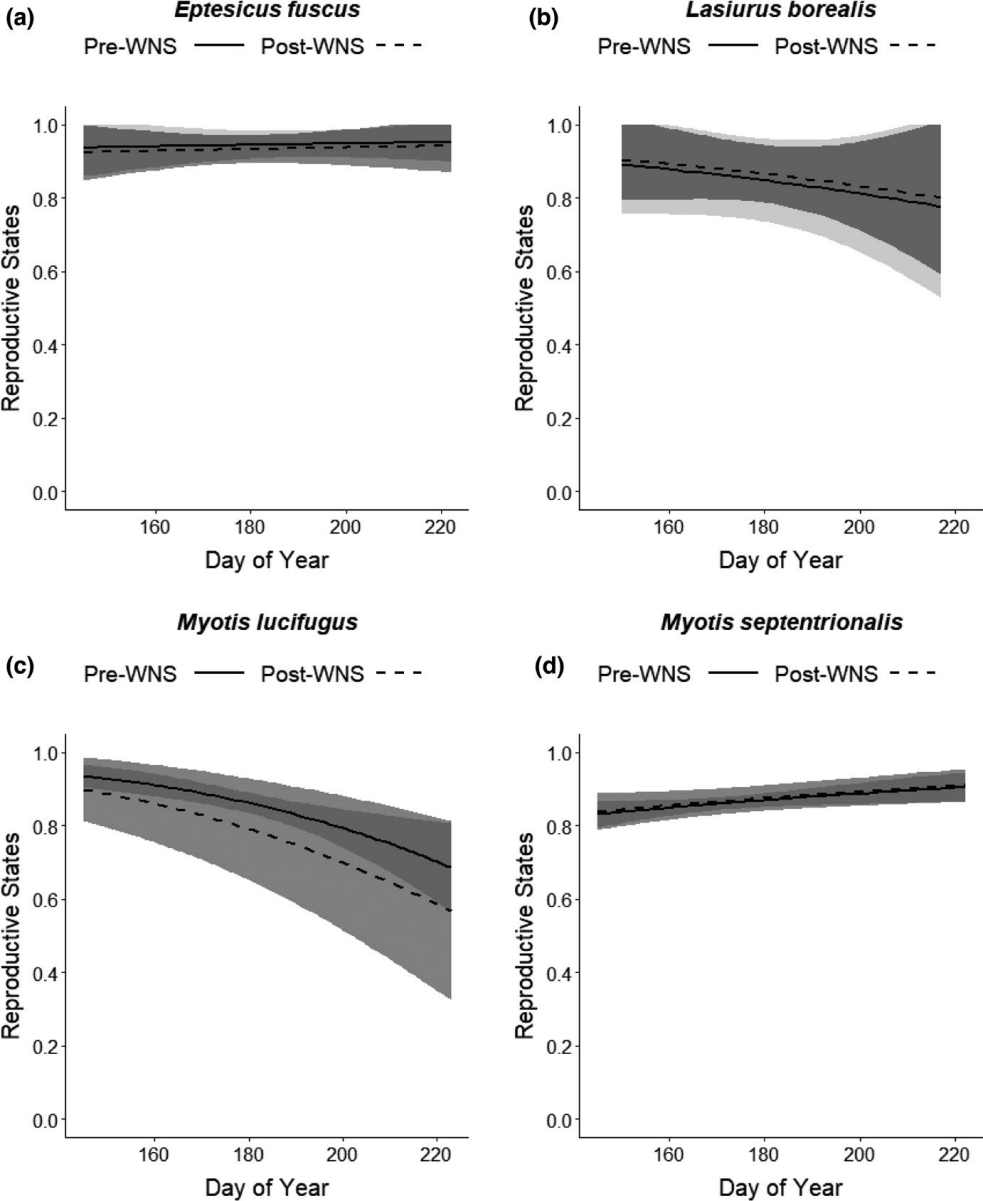
Estimated impacts of white‐nose syndrome (WNS) on probability of being in two states, nonreproductive or reproductive (all reproductive states included), for adult female bats in the Monongahela National Forest (MNF), West Virginia, USA, including (a) *Eptesicus fuscus* (big brown bat; *n* = 150 pre‐WNS and 165 post‐WNS), (b) *Lasiurus borealis* (eastern red bat; *n* = 39 pre‐WNS and 73 post‐WNS), (c) *Myotis lucifugus* (little brown bat; *n* = 599 pre‐WNS and 33 post‐WNS), and (d) *Myotis septentrionalis* (northern long‐eared bat; *n* = 1,589 pre‐WNS and 356 post‐WNS). We used logistic regression for the two‐state models, with observation years categorized as pre‐WNS (2003–2008) and post‐WNS (2011–2019), and day of year modeled as a continuous variable. The WNS invasion status predictor was not strongly supported for any of the species

**FIGURE 6:**
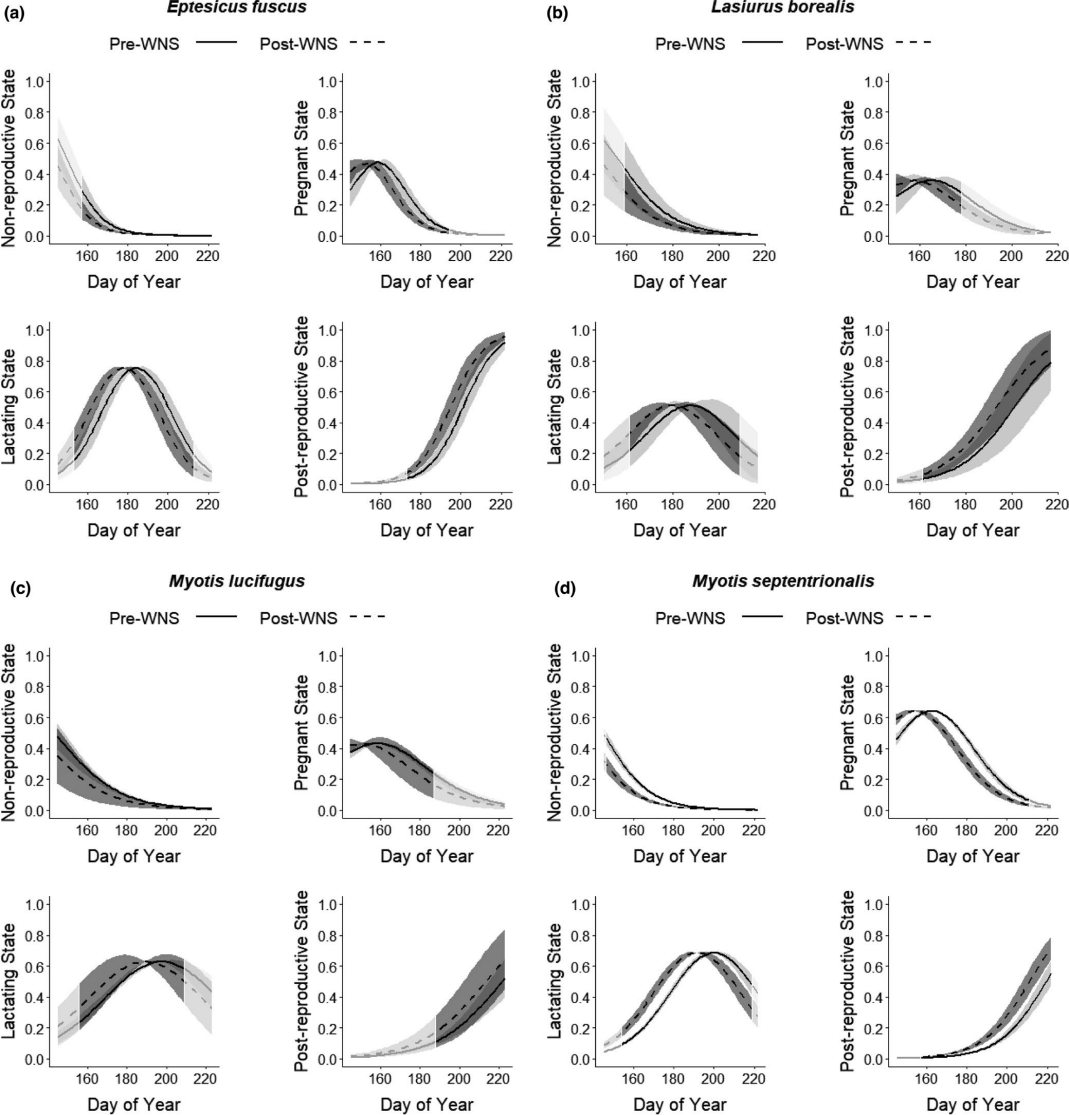
Estimated impacts of white‐nose syndrome (WNS) on probability of being in four states (nonreproductive, pregnant, lactating, or postreproductive) for adult female bats in the Monongahela National Forest (MNF), West Virginia, USA, including (a) *Eptesicus fuscus* (big brown bat; *n* = 150 pre‐WNS and 165 post‐WNS), (b) *Lasiurus borealis* (eastern red bat; *n* = 39 pre‐WNS and 73 post‐WNS), (c) *Myotis lucifugus* (little brown bat; *n* = 599 pre‐WNS and 33 post‐WNS), and (d) *Myotis septentrionalis* (northern long‐eared bat; *n* = 1,589 pre‐WNS and 356 post‐WNS). We used ordinal regression for the four‐state models, with observation years categorized as pre‐WNS (2003–2008) and post‐WNS (2011–2019), and day of year modeled as a continuous variable. For each state, the semiopaque portions of the plot display estimates beyond the earliest and latest day observed during sampling. The WNS invasion status predictor was strongly supported for *Eptesicus fuscus* and *Myotis septentrionalis*

Areas under the ROC curve (AUC) values ranged from 0.54 (*E*. *fuscus* and *M*. *septentrionalis*) to 0.61 (*M*. *lucifigus*), and thus, reproductive state results should be interpreted with caution for all species (Mandrekar, 2010). For the two‐state reproductive state models (nonreproductive or reproductive), invasion status was not a strongly supported predictor for any of the species modeled: *E*. *fuscus* (pre‐WNS β = 0.204, χ^2^_1_ = 0.174, *p* = .6768, Figure 5a); *L. borealis* (pre‐WNS β = −0.142, χ^2^_1_ = 0.064, *p* =.801, Figure 5b); *M*. *lucifugus* (pre‐WNS β = 0.507, χ^2^_1_ = 1.195, *p* = .274, Figure 5c); and *M*. *septentrionalis* (pre‐WNS β = −0.050, χ^2^_1_ = 0.087, *p* = .768, Figure 5d). While invasion status was not strongly supported for *M*. *lucifugus*, likely due to the small post‐WNS sample size [*n* = 33], the proportion of reproductively active females was lower post‐WNS (Figure 5c). For the four‐state reproductive state models, invasion status was a strongly supported predictor for *E*. *fuscus* (χ^2^_1_ = 8.663, *p* = .003, Figure 6a) and *M. septentrionalis* (χ^2^_1_ = 36.885, *p* < .0001, Figure 6d), but not for *L. borealis* (χ^2^_1_ = 3.225, *p* = .073, Figure 6b) or *M. lucifugus* (χ^2^_1_ = 1.879, *p* = .171, Figure 6c). The modeled response to WNS indicated that both *E*. *fuscus* and *M*. *septentrionalis* exhibited a post‐invasion shift to earlier reproduction (Figure 6a,d).

**FIGURE 7:**
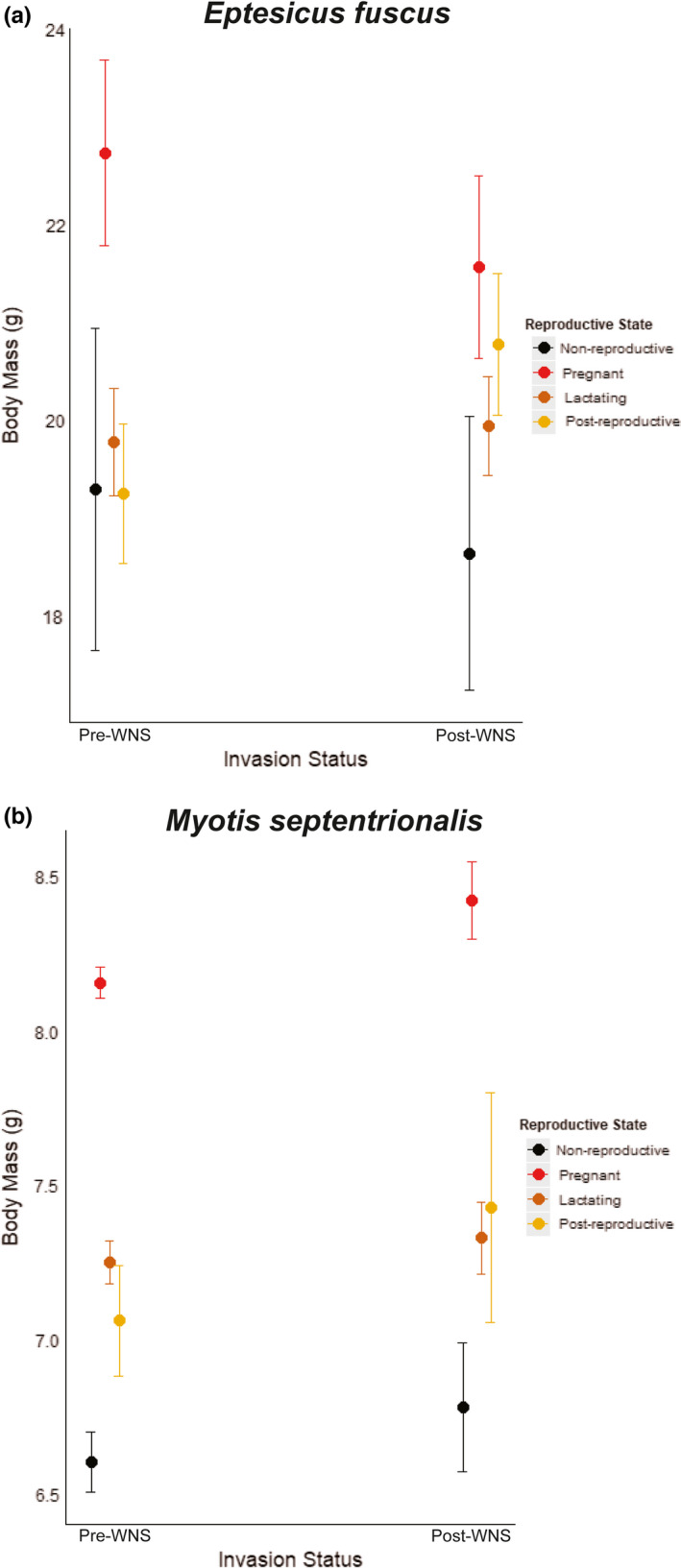
Estimated impacts of white‐nose syndrome (WNS) on body mass of female (a) *Eptesicus fuscus* (big brown bat; *n* = 145 pre‐WNS and 162 post‐WNS) and (b) *Myotis septentrionalis* (northern long‐eared bat; *n* = 1,543 pre‐WNS and 353 post‐WNS) in the Monongahela National Forest (MNF), West Virginia, USA. We used linear regressions with a Gaussian distribution, with observation years categorized as pre‐WNS (2003–2008) and post‐WNS (2011–2019), and an interaction effect between WNS invasion status (pre and post) and reproductive state (nonreproductive, pregnant, lactating, and postreproductive). Points represent model‐predicted body mass values and bars represent 95% confidence intervals

For body mass, invasion status had a supported interaction effect with reproductive state for *E*. *fuscus* (χ^2^_1_ = 12.155, *p* = .016) and *M*. *septentrionalis* (χ^2^_1_ = 21.552, *p* < .001), but not for *L. borealis* (χ^2^_2_ = 4.240, *p* = .374) or *M*. *lucifugus* (χ^2^_2_ = 6.227, *p* = .183). The *E*. *fuscus* model estimated that postinvasion body mass was lower for the pregnant and nonreproductive states, and higher for the lactating and postreproductive states (Figure 7; adjusted *r* ^2^ = 0.133). The *M*. *septentrionalis* model estimated that postinvasion body mass was higher for all reproductive states (Figure 7; adjusted *r* ^2^ = 0.133). However, 95% confidence intervals for interaction effect coefficients overlapped 0 (Table 1), indicating substantial variation in body mass among individuals.

**TABLE 1:** Coefficient estimates (β) and 95% confidence intervals (CI) for effects of white‐nose syndrome (WNS; pre [2003–2008] and post [2011–2019]) on body mass of female *Eptesicus fuscus* (big brown bat) and *Myotis septentrionalis* (northern long‐eared bat) in the Monongahela National Forest (MNF), West Virginia, USA. Models include an interaction effect between WNS invasion status and reproductive state (nonreproductive, pregnant, lactating, and postreproductive). The intercept represents predicted body mass (g) for the pre‐WNS nonreproductive state, and coefficients represent predicted change in body mass from the intercept for each parameter and interaction. Based on likelihood‐ratio tests, the WNS predictor was supported for both species, but WNS‐associated coefficient CI’s overlapped 0. Coefficient estimates with a CI that did not overlap 0 are bolded

Species	Parameter	β	95% CI
*Eptesicus fuscus*	Intercept	19.300	17.651 – 20.949
	Post‐WNS	−0.655	−2.822 – 1.513
	Pregnant	**3.438**	1.533 – 5.342
	Lactating	0.483	−1.256 – 2.223
	Postreproductive	−0.043	−1.842 – 1.756
	Post‐WNS × Pregnant	−0.511	−3.055 – 2.033
	Post‐WNS × Lactating	0.821	−1.472 – 3.114
	Post‐WNS × Postreproductive	2.178	−0.219 – 4.575
*Myotis septentrionalis*	Intercept	6.600	6.501 – 6.698
	Post‐WNS	0.178	−0.054 – 0.409
	Pregnant	**1.559**	1.448 – 1.670
	Lactating	**0.650**	0.529 – 0.771
	Postreproductive	**0.460**	0.256 – 0.664
	Post‐WNS × Pregnant	0.089	−0.179 – 0.357
	Post‐WNS × Lactating	−0.098	−0.367 – 0.170
	Post‐WNS × Postreproductive	0.190	−0.285 – 0.665

## 4 DISCUSSION

### 4.1 Bat community

Changes to bat populations following the onset of WNS were characterized by dramatic decreases in species that had previously comprised the largest portion of the bat community in the study area, particularly *M*. *septentrionalis* and *M*. *lucifugus*. These small myotids, together with *P*. *subflavu*s, had accounted for an average of 76.7% of bats captured each summer across the MNF from 2003 to 2008; however, counts of each of those species declined precipitously post‐WNS, together accounting for only 11.5% of captures by 2019. It is worth noting that a more subtle downward trend was observed for those species immediately preceding the first documentation of WNS in a cave in West Virginia in 2009 (Figure 3), which suggests the possibility that *Pd* may have been present but undetected in unmonitored hibernacula prior to formal discovery. *L. borealis* and *E*. *fuscus* both increased in relative abundance following WNS, going from a combined average of 18.7% of total captures pre‐WNS to 68.9% in 2019 (Figure 4). This dramatic shift in MNF bat community composition has been noted in similar studies of summer bat communities across eastern North America where species most vulnerable to WNS have experienced dramatic declines (e.g., Faure‐Lacroix et al., 2020; Jachowski et al., 2014; Moosman et al., 2013; O’Keefe et al., 2019; Pettit & O’Keefe, 2017) and is similar to patterns seen in hibernacula. Our study includes a longer continuous time frame of monitoring both pre‐ and post‐WNS than previous studies, however, allowing local bat communities an extended period of time to adjust to the presence of WNS and providing greater insight into what may be a new normal for community composition and relative abundance of bat species across the MNF post‐WNS. Interestingly, while the species noted above showed different patterns pre‐ and post‐WNS, other species (e.g., *M*. *leibii* and *C.t*. *virginianus*) were relatively unaffected post‐WNS (Figure 4). Counts from the 11 known maternity colonies of *C.t*. *virginianus* showed a steady increase of approximately 4%–5% annually throughout the 17‐year monitoring period prior to 2019 (WVDNR, unpublished data).

Our study documented increases in relative abundance of *E*. *fuscus* and *L. borealis* immediately post‐WNS. *L. borealis* numbers decreased to levels similar to pre‐WNS counts after 2013, but both presence and counts of *E*. *fuscus* continued to increase throughout the study period. It is possible that the consistent post‐WNS increase in *E*. *fuscus* seen in our study is an artifact of sampling space, with *E*. *fuscus* changing their use of foraging habitat near nets or being more readily captured as a result of the decrease in WNS‐affected bats using airspace around the nets (Pettit & O’Keefe, 2017). However, if that is the case, we might have expected to see increases in *L. borealis* captures continue as well.

If not a sampling artifact, the increase in *E*. *fuscus* in our study area could be indicative of a competitive release and niche expansion (related to either increased foraging or roosting opportunities) in response to the rapid loss of several species that had previously comprised the majority of the bat community. From the foraging perspective, *E*. *fuscus* may have been able to capitalize on the loss of smaller myotids and the release of interspecific foraging competition during summer on the MNF. Although studies have shown significant differences in the diets of the smaller myotids most affected by WNS such as *M*. *lucifugus* and *M*. *septentrionalis*, as compared to more WNS‐resistant species such as *E*. *fuscus* and *L. borealis* (Clare et al., 2009, 2011; Dodd et al., 2012; Wray et al., 2018), competitive release associated with the dramatic declines of the WNS‐vulnerable species could result in a change in diet of more plastic WNS‐resistant species (Alberdi et al., 2019; Jachowski et al., 2014). In Southern Ontario, where acoustic activity of *E*. *fuscus* increased significantly as *M*. *lucifugus* declined, stomach samples of *E*. *fuscus* showed an increase in diet richness post‐WNS, including an increase in insect species in common with *M*. *lucifugus* (from 37 to 58; Morningstar et al., 2019). Clare et al. (2014) also found an increase in the overlap of insect prey species between *E*. *fuscus* and *M*. *lucifugus* post‐WNS.

Similarly, from the roosting perspective, the increase in *E*. *fuscus* summer numbers could be related to an increase in the availability of high‐quality summer roosts as *M*. *lucifugus* populations declined. The two species use roosts with analogous conditions in West Virginia (e.g., attics and similar structures), and replacement of *M*. *lucifugus* colonies with *E*. *fuscus* colonies in buildings has been reported elsewhere (Cope et al., 1991). Since the post‐WNS decline of *M*. *lucifugus* populations in Pennsylvania, state biologists have also documented shifts to *E*. *fuscus* colonies in 6 maternity roost sites that previously supported large *M*. *lucifugus* colonies (G. Turner, Personal communication). Future research efforts examining conditions and relative use of maternity roosts by these and other species would be useful to better inform our understanding of optimal maternity colony conditions and potential competition across species.

### 4.2 Reproductive state and body mass

*Myotis lucifugus* females in our study area were less likely to be reproductively active post‐WNS (Figure 5c), though not significantly so (likely due to small post‐WNS sample sizes), suggesting that WNS‐related stressors may have an adverse effect on the reproductive capacity of this species. *M. lucifugus* and other WNS‐affected species that survive *Pd* exposure are faced with extreme energetic imbalance associated with healing of wing damage and reduced foraging efficiency (Fuller et al., 2020; Meierhofer et al., 2018). Captive *M*. *lucifugus* that survived *Pd* infection in hibernation had increased wing tissue damage and elevated mass‐specific resting metabolic rate compared with *Pd*‐uninfected bats, suggesting greater energetic costs during spring in WNS survivors (Meierhofer et al., 2018). In addition, chronic stress levels in free‐ranging bats indicate that physiological consequences of *Pd* infection persist even after infected bats have emerged from hibernation and recovered from wing damage and other outward signs of disease (Davy et al., 2017). WNS survivors faced with increased energetic costs associated with wing tissue damage repair and other aspects of disease recovery also may be unable to forage as efficiently as they normally would due to impaired flight performance (Fuller et al., 2020). Such Pd survivors, with increased energetic demands and decreased foraging efficiency, may be unable to expend the additional high energetic costs associated with successful rearing of young.

Although *M*. *lucifugus* populations in the northeast may be evolving a resistance to *Pd* mortality during hibernation (Frank et al., 2019; Langwig et al., 2017), populations that fail to meet their pre‐WNS reproductive potential likely will continue to decline. Studies of *M*. *lucifugus* maternity colonies in the vicinity of the WNS epicenter show successful reproduction and colony growth, with increasing survival probabilities post‐WNS (Dobony & Johnson, 2018; Ineson, 2020). However, our results suggest that the proportion of reproductively active females may be lower for WNS‐affected *M*. *lucifugus* in West Virginia post‐WNS. The timing of our long‐term surveys precludes the ability to detect changes in the proportion of juveniles across years, but caution must also be taken in assessing reproductive rates from maternity colonies and extrapolating to populations (Barclay et al., 2004). Thus, a combination of summer mist‐net monitoring combined with maternity colony studies may be required to gain a fuller understanding of the reproductive fitness of recovering *M*. *lucifugus* populations in a given area.

As compared to the laboratory and field studies that have been conducted on *M*. *lucifugus*, including those examining the impacts of WNS, there is a paucity of data regarding the impacts of WNS on reproduction or body condition of *M*. *septentrionalis*. Our results indicated the proportion of reproductively active *M*. *septentrionalis* females was nearly identical pre‐ and post‐WNS, suggesting that WNS‐affected adult females of the two species may be dealing with the physiological impacts of *Pd* infection in different ways or may be employing different strategies to cope with impacts of the disease. We also did not observe an adverse impact to body condition for reproductive *M*. *septentrionalis* post‐WNS. Rather, a nonsignificant increase in body mass for adult female *M*. *septentrionalis* in all reproductive states was observed post‐WNS, suggesting that the physiological costs of reproduction for females was not having a greater impact on their body condition post‐WNS. This finding and the lack of a decrease in reproductively active females are contrary to our expectation that the physiological cost of WNS recovery would have an adverse effect on survivors as reflected in reproduction and/or body condition. However, our study was not designed to track changes in juvenile survival or condition across years, nor whether WNS‐affected females that produced young survived to reproduce the following spring, so we cannot directly address reproductive fitness for *M*. *septentrionalis* post‐WNS. It is also possible that persisting *M*. *septentrionalis* populations in West Virginia may be hibernating in locations other than the large hibernacula that support most *M*. *lucifugus* overwintering in the area and thus may be avoiding the highest levels of *Pd* exposure. Hibernation sites housing small numbers of WNS‐vulnerable bat species may have high survival rates despite WNS, and locations with many such small hibernacula could help to maintain persistence of a population at low levels post‐WNS (Perry & Jordan, 2020). Additional research is needed to better understand the differential impacts that WNS may have on the reproductive fitness of *M*. *septentrionalis* and *M*. *lucifugus* or other WNS‐affected species. Identification of overwintering locations for successfully reproducing *M*. *septentrionalis* females also could help to illuminate potential reasons for the interspecific differences observed in this study.

While we did not observe a decrease in the proportion of reproductively active females post‐WNS, our data did indicate a significant shift in timing to earlier reproduction (approximately 6 days) for *M*. *septentrionalis* and a similar shift for *E. fuscus* post‐WNS (approximately 5 days). Similarly, a long‐term study in northern New England detected an advance of 6–10 days in reproductive timing for *M*. *lucifugus* since the onset of WNS (Ineson, 2020). Long‐term monitoring of Mexican free‐tailed bats (*Tadarida brasiliensis mexicana*) at Bracken Cave also showed a 2‐week advance in timing for spring migration and the summer reproductive cycle over a 22‐year study period (Stepanian & Wainwright, 2018). Climate change is likely a cause for at least some of these observed changes in phenology. However, WNS also could be playing a role in these shifts for WNS‐affected species. Ineson (2020) also found some evidence for an advance in reproductive timing for *M*. *lucifugus* post‐WNS independent of climate, though both spring temperature and precipitation also contributed to changes in reproductive phenology.

Earlier reproduction could be beneficial for WNS survivors that do attempt to raise pups as young born earlier in the summer may have a higher probability of surviving their first year (Frick et al., 2010). However, environmental changes associated with global climate change in some regions (e.g., higher precipitation rates during foraging hours or lower precipitation rates leading to fewer water sources, decreases or changes in phenology of insect resources, decreases in optimal or suitable roosts, unsuitably hot or cold temperatures) also could exacerbate WNS‐related bat population declines (Hayes & Adams, 2017). Reductions in the availability of water across landscapes, such as that associated with the warmer and dryer conditions projected across much of Western North America, may result in declines in reproductive success of bat populations (Adams, 2010). Models of suitable maternity colony habitat for *M*. *sodalis* based on projected climate change forecast a decline in aerial extent and a concentration of colonies in the northeastern U.S. and Appalachian Mountains (Loeb & Winters, 2013). Regional or more local distributional shifts might also be expected for other species, making management of roosting habitat even more important in areas where climatic conditions will remain or become suitable under future climate change scenarios. Further research is needed to test projections and expand our understanding of climate change impacts on bat species and populations at varying spatial scales, particularly those already in decline as a result of WNS, loss of habitat, and other stressors.

### 4.3 Management implications

Given the higher physiological demands of *Pd*‐infected species in spring and early summer, the ability to quickly heal wing damage and put on fat would likely enhance survival of emerging individuals as well as the likelihood of maintaining energy reserves sufficient to support successful reproduction. Conservation and management for WNS‐affected bats have long been focused on improving survival during hibernation, a clearly critical need to stem the devastating mortality rates seen in newly *Pd*‐infected populations. The importance of conservation and management of habitats used during the active seasons (spring, summer, and fall) has received less attention despite the importance of successful reproduction to recovery of species, particularly for remnant post‐WNS populations.

Habitat management focused on helping WNS‐affected bats achieve energy balance upon emergence from hibernacula could be critical (Fuller et al., 2020). Active‐season habitat management includes foraging habitat as well as roosting habitat and can range from sound forest management practices to providing roosts that minimize thermoregulatory costs of recovering bats (Wilcox & Willis, 2016). While habitat preferences are not uniform across North American bat species, some forest habitat features are beneficial for most WNS‐affected species, and guidance is available to help land managers meet those habitat needs at a variety of spatial scales (e.g., Johnson et al., 2018; Silvis et al., 2016). Given the need to better understand the impacts of WNS during the nonhibernation season and the eagerness of land managers to proactively help in the fight to save WNS‐affected bats, research and management focused on posthibernation recovery and enhancing reproductive success in WNS‐affected bats could yield population‐level benefits, especially in areas where WNS has long been established and the focus is on population recovery.

## CONFLICT OF INTEREST

None declared.

## AUTHOR CONTRIBUTIONS

**Catherine Johnson:** Conceptualization (equal); Data curation (equal); Investigation (equal); Methodology (equal); Writing‐original draft (lead); Writing‐review & editing (equal). **Donald J. Brown:** Conceptualization (equal); Data curation (supporting); Formal analysis (lead); Visualization (lead); Writing‐review & editing (equal). **Chris Sanders:** Data curation (equal); Investigation (equal); Methodology (equal); Writing‐review & editing (supporting). **Craig W. Stihler:** Conceptualization (supporting); Investigation (supporting); Writing‐review & editing (equal).

## Data Availability

The data used in this study are archived in the Dryad data repository: https://doi.org/10.5061/dryad.r4xgxd2cv.
